# Electro-osmotic flow of biological fluid in divergent channel: drug therapy in compressed capillaries

**DOI:** 10.1038/s41598-021-03087-0

**Published:** 2021-12-08

**Authors:** Yun-Jie Xu, Mubbashar Nazeer, Farooq Hussain, M. Ijaz Khan, M. K. Hameed, Nehad Ali Shah, Jae Dong Chung

**Affiliations:** 1 School of Engineering, Huzhou University, Huzhou, 313000 People’s Republic of China; 2 Department of Mathematics, Institute of Arts and Sciences, Government College University Faisalabad Chiniot Campus, Chiniot, 35400 Pakistan; 3 Department of Mathematical Sciences (FBAS), BUITEMS, Quetta, 87300 Pakistan; 4 Department of Mathematics and Statistics, Riphah International University, I-14, Islamabad, 44000 Pakistan; 5 Nonlinear Analysis and Applied Mathematics (NAAM) Research Group, Department of Mathematics, Faculty of Science, King Abdulaziz University, P.O. Box 80257, Jidda, 21589 Saudi Arabia; 6 Department of Mathematics, Riphah International University Faisalabad Campus, Faisalabad, 38000 Pakistan; 7 Department of Mechanical Engineering, Sejong University, Seoul, 05006 Korea; 8 Department of Mathematics, Lahore Leads University, Lahore, Pakistan

**Keywords:** Engineering, Mathematics and computing

## Abstract

The multi-phase flow of non-Newtonian through a divergent channel is studied in this article. Jeffrey fluid is considered as the base liquid and tiny gold particles for the two-phase suspension. Application of external electric field parallel to complicated capillary with net surface charge density causes the bulk motion of the bi-phase fluid. In addition to, electro-osmotic flow with heat transfer, the simultaneous effects of viscous dissipation and nonlinear thermal radiation have also been incorporated. Finally, cumbersome mathematical manipulation yields a closed-form solution to the nonlinear differential equations. Parametric study reveals that more thermal energy is contributed in response to Brinkman number which significantly assists gold particles to more heat attain high temperature, as the remedy for compressed or swollen capillaries/arteries.

## Introduction

Electro-osmotic flow^1,2^ is mainly concerned with the application of electric field which is applied externally. Electricity/electric fields interact to charge density acting in the transverse direction, to generate the motion of bulk fluid in the direction, parallel to the applied electric fields. Nazeer et al.^3^ provide an analytical solution for the non-Newtonian fluid flow under the effects of applied electric fields. In^4^, electro-osmotic flow of two-phase Newtonian fluid is investigated through three different configurations. The study reveals that jet-shaped geometry is quite suitable for multiphase flows. Ellahi et al.^5^ incorporate the lubricating walls on the symmetric channels for analyzing electro-osmotic flows of a Newtonian fluid. Mekheimer et al.^6^ are relevant to electro-osmotic flows of two types of nanofluids suspended with gold and copper particles. Blood is used as the base fluid to form the physiological nanofluids under the effects of entropy generation. Saleem et al.^7^ performed the symbolic software to obtain the exact solution of the electro-osmotic flow of complex rheological fluid.

Non-Newtonian fluid has vast application in daily life from geographical flow to industrial and mechanical flow. Jeffrey fluid is also one of the non-Newtonian fluids which linearly relate relaxation time to retardation time. Firdous et al.^8^ discussed the simultaneous influences of magnetic fields on the Jeffrey fluid flow. To expedite the flow and heat transfer different kinds of slip conditions such as momentum slip and thermal slip boundary conditions are imposed.

An analytical prediction of multiple solutions for MHD Jeffrey–Hamel flow and heat transfer utilizing KKL nanofluid model is reported by Rana et al.^9^. Ahmed et al.^10^ consider the nanofluid flow of Jeffrey fluid, due to stretching surface with the application of external heating effects. Ellahi et al.^11,12^ applied separation of variables to analyze the two-dimensional flow of Jeffrey fluid through a rectangular duct. An exact solution explicitly elaborates the magnetohydrodynamics (MHD) and lubrication effects on the parallel walls on the peristaltic transport of Jeffrey fluid respectively with and without porosity.

Multi-phase flow is a ubiquitous phenomenon such as from rain to rivers flowing through valleys, from the extraction of crude oils to immiscible-liquid mixtures, from different kinds of chemical and pharmaceutical processes to biological fluid transporting in the human body, etc., are some common examples of multiphase flows^13–17^. Ellahi et al*.*^18^ formulate four different kinds of multiphase flows suspended with Newtonian fluids. They applied a thin shinning sheet/layer on the rotating disk, by using gold and silver particles. They inferred that ethanol suspension with gold particles yields a perfect coating on any rotating surface. Nazeer et al.^19^ obtained an approximate solution for two different kinds of multiphase flows. Suspensions are formed by considering Third-grade fluid as the base liquid while, Hafnium and crystal particles are considered. The gravitational force causes the flow of an MHD multiphase flow through an inclined channel. In^16^, Couette flow of Couple stress fluid is simulated. The magnetized moving upper wall of the channel drives the two-phase flow. The heating effects at the boundary attenuates the shear thickening effects. Zeeshan et al.^20^ have applied numerical techniques for a free-stream flow on an inclined sheet. Range–Kutta method with shooting technique is applied to obtain a numerical solution of nonlinear differential equations. Paul et al.^21^ worked on modeling of industrial particle and multiphase flows using combinations of DEM for free surface fluid-particle flows.

Internal flows through closed channels change the hydrodynamic structure of the flow. There can be rapid dynamical changes in the internal flows, for the uniform channels with the compressed portion or a divergent configuration. Zheng et al*.*^22^ brought convergent-divergent slit ribs to improve internal cooling. They observed that there is a vivid thermal enhancement working with small-angle trapezoidal slits that increase heat transfer. Mekheimer et al.^23^ use gold nanoparticles as drug agents for therapy and, suggest that gold nanoparticles effectively contribute to drug delivery. Intrauterine particle–fluid motion through a compliant asymmetric tapered channel with heat transfer is reported by Bhatti et al.^24^. In^25,26^, nano-blood flows through catharized tapered arteries are reported. Jeffrey fluid is treated as the physiological fluid by using gold nano-particles work as the remedy. Some important studies are listed in the Refs.^27–31^.

In view of fore-going literature, it is evident that no attention has been paid towards the two-phase flow of Jeffrey fluid with heat transfer in a convergent channel. Additional contributions of viscous dissipation and nonlinear radiative flux are taken into account, as well. The electro-osmotic flow of Jeffrey fluid suspended with gold particles is an innovative concept which addresses the blood transport through compressed/swollen capillaries or arteries.

## Mathematical model

**Figure 1:**
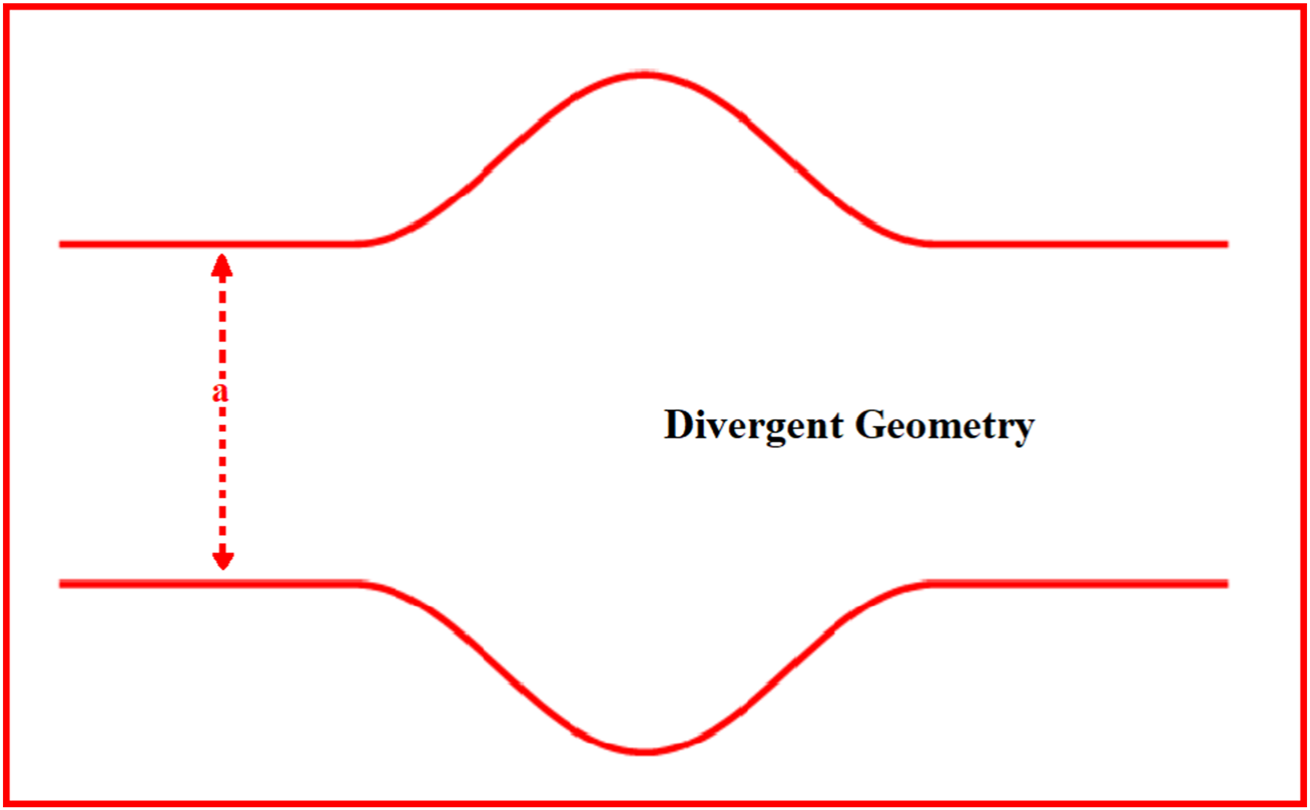
Divergent geometry.

Consider a steady two-phase flow of a non-Newtonian fluid with heat transfer in a divergent channel as shown in Fig. 1. The multiphase suspension is composed of Jeffrey fluid as the base liquid suspended with tiny size confined by gold particles. Let $$V_{f} = \left[ {u_{f} \left( {\overset{\lower0.5em\hbox{$\smash{\scriptscriptstyle\frown}$}}{\zeta } ,{\overset{\lower0.5em\hbox{$\smash{\scriptscriptstyle\frown}$}}{\eta } }} \right){ , }0{ , }0} \right]$$ and $$V_{p} = \left[ {u_{p} \left( {\overset{\lower0.5em\hbox{$\smash{\scriptscriptstyle\frown}$}}{\zeta } ,{\overset{\lower0.5em\hbox{$\smash{\scriptscriptstyle\frown}$}}{\eta } }} \right){ , }0 \, , \, 0} \right]$$ are the velocity profiles of liquid and particle phases, respectively.

1 $$ H_{G} (\overset{\lower0.5em\hbox{$\smash{\scriptscriptstyle\frown}$}}{\zeta } ) = \left\{ {\begin{array}{*{20}c} {a_{1} - a_{2} \sin^{2} \left( {\frac{{\pi \overset{\lower0.5em\hbox{$\smash{\scriptscriptstyle\frown}$}}{\zeta } }}{\uplambda }} \right)} & {{\text{When }}\frac{{\uplambda 11}}{7}{ < }\overset{\lower0.5em\hbox{$\smash{\scriptscriptstyle\frown}$}}{\zeta } { < }\frac{{{\uplambda  33}}}{7},} \\ {0.5a_{1} ;} & {{\text{Otherwise}}{.}} \\ \end{array} } \right. $$

For the fluid phase,

2 $$ \nabla .\user2{\overset{\lower0.5em\hbox{$\smash{\scriptscriptstyle\frown}$}}{V} }_{f} = 0, $$

3 $$ \rho_{f} {(1 - }{\text{C}}{)}\frac{{D\;\user2{\overset{\lower0.5em\hbox{$\smash{\scriptscriptstyle\frown}$}}{V} }_{f} }}{D\;t}{ = - (1 - }{\text{C}}{)}\nabla \cdot \overset{\lower0.5em\hbox{$\smash{\scriptscriptstyle\frown}$}}{p} { + }\frac{{{(1 - }{\text{C}}{)}}}{{1 + \lambda_{1} }}\nabla \cdot {\varvec{S}} + SC\left( {\user2{\overset{\lower0.5em\hbox{$\smash{\scriptscriptstyle\frown}$}}{V} }_{p} - \user2{\overset{\lower0.5em\hbox{$\smash{\scriptscriptstyle\frown}$}}{V} }_{f} } \right){ + \sigma {\rm B}}_{o}^{2} \overset{\lower0.5em\hbox{$\smash{\scriptscriptstyle\frown}$}}{u}_{f} + \rho_{f} g + \nabla^{2}\Phi \overrightarrow {{E_{{\overset{\lower0.5em\hbox{$\smash{\scriptscriptstyle\frown}$}}{\zeta } }} }} . $$

where the stress tensor “***S***” is defined as^32,33^

4 $$ {\varvec{S}} = \frac{{\mu_{s} }}{{1 + \lambda_{1} }}(\dot{r} + \lambda_{2} \ddot{r}), $$

5 $$ {\dot{\text{r}}} = {\kern 1pt} \, {\mathbf{L}}{ + }{\mathbf{L}}^{{\text{T}}} , $$

6 $$ \ddot{r} = \frac{{d\dot{r}}}{dt}. $$

For the particle phase,

7 $$ \nabla .\user2{\overset{\lower0.5em\hbox{$\smash{\scriptscriptstyle\frown}$}}{V} }_{p} = 0, $$

8 $$ \rho_{p} {\text{C}}\frac{{D\;\user2{\overset{\lower0.5em\hbox{$\smash{\scriptscriptstyle\frown}$}}{V} }_{p} }}{D\;t}{ = - }{\text{C}}\;\nabla \cdot \overset{\lower0.5em\hbox{$\smash{\scriptscriptstyle\frown}$}}{p} { + }{\text{C}}S\left( {\user2{\overset{\lower0.5em\hbox{$\smash{\scriptscriptstyle\frown}$}}{V} }_{f} - \user2{\overset{\lower0.5em\hbox{$\smash{\scriptscriptstyle\frown}$}}{V} }_{p} } \right). $$

The heat equation for the multiphase flow under the consideration of the thermal radiation and viscous dissipation is defined by

9 $$ \rho_{f} (C_{p} )_{f} \frac{{D\overset{\lower0.5em\hbox{$\smash{\scriptscriptstyle\frown}$}}{T} }}{Dt} = \nabla \cdot k\nabla \overset{\lower0.5em\hbox{$\smash{\scriptscriptstyle\frown}$}}{T} + \mu_{s} \phi - \left( {\frac{{\partial q_{{r\overset{\lower0.5em\hbox{$\smash{\scriptscriptstyle\frown}$}}{\zeta } }} }}{{\partial \overset{\lower0.5em\hbox{$\smash{\scriptscriptstyle\frown}$}}{\zeta } }} + \frac{{\partial q_{{r\overset{\lower0.5em\hbox{$\smash{\scriptscriptstyle\frown}$}}{\eta } }} }}{{\partial \overset{\lower0.5em\hbox{$\smash{\scriptscriptstyle\frown}$}}{\eta } }}} \right). $$

The component form of Eqs. (2), (3), (7), (8) and (9) are given as

10 $$ \frac{{\partial \overset{\lower0.5em\hbox{$\smash{\scriptscriptstyle\frown}$}}{u}_{f} }}{{\partial {\overset{\lower0.5em\hbox{$\smash{\scriptscriptstyle\frown}$}}{\xi } }}} + \frac{{\partial \overset{\lower0.5em\hbox{$\smash{\scriptscriptstyle\frown}$}}{v}_{f} }}{{\partial {\overset{\lower0.5em\hbox{$\smash{\scriptscriptstyle\frown}$}}{\eta } }}} = 0, $$

11 $$ \left. \begin{gathered}\uprho _{f} {(1 - }{\text{C}}{)}\left[ {\overset{\lower0.5em\hbox{$\smash{\scriptscriptstyle\frown}$}}{u}_{f} \frac{{\partial \overset{\lower0.5em\hbox{$\smash{\scriptscriptstyle\frown}$}}{u}_{f} }}{{\partial \overset{\lower0.5em\hbox{$\smash{\scriptscriptstyle\frown}$}}{\zeta } }} + \overset{\lower0.5em\hbox{$\smash{\scriptscriptstyle\frown}$}}{v}_{f} \frac{{\partial \overset{\lower0.5em\hbox{$\smash{\scriptscriptstyle\frown}$}}{v}_{f} }}{{\partial {\overset{\lower0.5em\hbox{$\smash{\scriptscriptstyle\frown}$}}{\eta } }}}} \right]{ = - (1 - }{\text{C}}{)}\frac{{\partial \overset{\lower0.5em\hbox{$\smash{\scriptscriptstyle\frown}$}}{p} }}{{\partial \overset{\lower0.5em\hbox{$\smash{\scriptscriptstyle\frown}$}}{\zeta } }}{ + }\frac{{\mu_{s} }}{{1 + \lambda_{1} }}{(1 - }{\text{C}}{)} \hfill \\ \left[ {\frac{{\partial^{2} \overset{\lower0.5em\hbox{$\smash{\scriptscriptstyle\frown}$}}{u}_{f} }}{{\partial \overset{\lower0.5em\hbox{$\smash{\scriptscriptstyle\frown}$}}{\zeta }^{2} }} + \frac{{\partial^{2} \overset{\lower0.5em\hbox{$\smash{\scriptscriptstyle\frown}$}}{v}_{f} }}{{\partial {\overset{\lower0.5em\hbox{$\smash{\scriptscriptstyle\frown}$}}{\eta } }^{2} }}} \right]{\text{ + C}}S\left( {\overset{\lower0.5em\hbox{$\smash{\scriptscriptstyle\frown}$}}{u}_{p} - \overset{\lower0.5em\hbox{$\smash{\scriptscriptstyle\frown}$}}{u}_{f} } \right){ - \sigma {\rm B}}_{o}^{2} \overset{\lower0.5em\hbox{$\smash{\scriptscriptstyle\frown}$}}{u}_{f} + \left[ {\frac{{\partial^{2}\Phi }}{{\partial \overset{\lower0.5em\hbox{$\smash{\scriptscriptstyle\frown}$}}{\zeta }^{2} }} + \frac{{\partial^{2}\Phi }}{{\partial {\overset{\lower0.5em\hbox{$\smash{\scriptscriptstyle\frown}$}}{\eta } }^{2} }}} \right]\overrightarrow {{E_{{\overset{\lower0.5em\hbox{$\smash{\scriptscriptstyle\frown}$}}{\zeta } }} }} \hfill \\ \end{gathered} \right\}, $$

12 $$ \frac{{\partial \overset{\lower0.5em\hbox{$\smash{\scriptscriptstyle\frown}$}}{u}_{p} }}{{\partial \overset{\lower0.5em\hbox{$\smash{\scriptscriptstyle\frown}$}}{\zeta } }} + \frac{{\partial \overset{\lower0.5em\hbox{$\smash{\scriptscriptstyle\frown}$}}{v}_{p} }}{{\partial {\overset{\lower0.5em\hbox{$\smash{\scriptscriptstyle\frown}$}}{\eta } }}} = 0, $$

13 $$\uprho _{p} {\text{C}}\left[ {\overset{\lower0.5em\hbox{$\smash{\scriptscriptstyle\frown}$}}{u}_{p} \frac{{\partial \overset{\lower0.5em\hbox{$\smash{\scriptscriptstyle\frown}$}}{u}_{p} }}{{\partial \overset{\lower0.5em\hbox{$\smash{\scriptscriptstyle\frown}$}}{\zeta } }} + \overset{\lower0.5em\hbox{$\smash{\scriptscriptstyle\frown}$}}{v}_{p} \frac{{\partial \overset{\lower0.5em\hbox{$\smash{\scriptscriptstyle\frown}$}}{v}_{p} }}{{\partial {\overset{\lower0.5em\hbox{$\smash{\scriptscriptstyle\frown}$}}{\eta } }}}} \right]{ = - }{\text{C}}\frac{{\partial \overset{\lower0.5em\hbox{$\smash{\scriptscriptstyle\frown}$}}{p} }}{{\partial \overset{\lower0.5em\hbox{$\smash{\scriptscriptstyle\frown}$}}{\zeta } }}{ + }{\text{C}}S\left( {\overset{\lower0.5em\hbox{$\smash{\scriptscriptstyle\frown}$}}{u}_{f} - \overset{\lower0.5em\hbox{$\smash{\scriptscriptstyle\frown}$}}{u}_{p} } \right), $$

14 $$ \rho_{f} C_{p} \left( {\frac{\partial }{\partial t} + \frac{\partial }{{\partial \overset{\lower0.5em\hbox{$\smash{\scriptscriptstyle\frown}$}}{\zeta } }} + \frac{\partial }{{\partial \overset{\lower0.5em\hbox{$\smash{\scriptscriptstyle\frown}$}}{\eta } }}} \right)\overset{\lower0.5em\hbox{$\smash{\scriptscriptstyle\frown}$}}{T} = \frac{\partial }{{\partial \overset{\lower0.5em\hbox{$\smash{\scriptscriptstyle\frown}$}}{\eta } }}\left( {k\frac{{\partial \overset{\lower0.5em\hbox{$\smash{\scriptscriptstyle\frown}$}}{T} }}{{\partial \overset{\lower0.5em\hbox{$\smash{\scriptscriptstyle\frown}$}}{\eta } }}} \right) + \frac{{\mu_{s} }}{{1 - \lambda_{1} }}\left( {\frac{\partial u}{{\partial \overset{\lower0.5em\hbox{$\smash{\scriptscriptstyle\frown}$}}{\eta } }}} \right)^{2} - \frac{{16\sigma^{ * } }}{{3k^{ * } }}\left( {\frac{{\partial^{2} \overset{\lower0.5em\hbox{$\smash{\scriptscriptstyle\frown}$}}{T} }}{{\partial \overset{\lower0.5em\hbox{$\smash{\scriptscriptstyle\frown}$}}{\zeta }^{2} }} + \frac{{\partial^{2} \overset{\lower0.5em\hbox{$\smash{\scriptscriptstyle\frown}$}}{T} }}{{\partial \overset{\lower0.5em\hbox{$\smash{\scriptscriptstyle\frown}$}}{\eta }^{2} }}} \right). $$

The dimensional form of the boundary are defined by

1. 15 $$ \overset{\lower0.5em\hbox{$\smash{\scriptscriptstyle\frown}$}}{u}_{f} = \overset{\lower0.5em\hbox{$\smash{\scriptscriptstyle\frown}$}}{u}_{{f_{At \, the \, wall} }} ;\quad When \, {\overset{\lower0.5em\hbox{$\smash{\scriptscriptstyle\frown}$}}{\eta } } = H_{G} (\overset{\lower0.5em\hbox{$\smash{\scriptscriptstyle\frown}$}}{\zeta } ), $$
2. 16 $$ \overset{\lower0.5em\hbox{$\smash{\scriptscriptstyle\frown}$}}{u}_{f} = \overset{\lower0.5em\hbox{$\smash{\scriptscriptstyle\frown}$}}{u}_{{f_{At \, the \, wall} }} ;\quad When \, {\overset{\lower0.5em\hbox{$\smash{\scriptscriptstyle\frown}$}}{\eta } } = - H_{G} (\overset{\lower0.5em\hbox{$\smash{\scriptscriptstyle\frown}$}}{\zeta } ). $$

The following transformation is used to get the non-dimensional form of the above equations

17 $$  \left. \begin{gathered}   {\bar{\xi }} = \frac{{\overset{\lower0.5em\hbox{$\smash{\scriptscriptstyle\frown}$}}{\zeta } }}{{\lambda }},{\bar{\eta }}{{ = }}\frac{{{\overset{\lower0.5em\hbox{$\smash{\scriptscriptstyle\frown}$}}{\eta } }}}{{a_{1} }},{{  \bar{\text{u}}}}_{f} {{ = }}\frac{{{{\overset{\lower0.5em\hbox{$\smash{\scriptscriptstyle\frown}$}}{u} }}_{f} }}{{U_{o} }},{{   \bar{\text{u}}}}_{p} {{ = }}\frac{{{{\overset{\lower0.5em\hbox{$\smash{\scriptscriptstyle\frown}$}}{u} }}_{p} }}{{U_{o} }},\bar{v}_{f} {{ = }}\frac{{\overset{\lower0.5em\hbox{$\smash{\scriptscriptstyle\frown}$}}{v} _{f} }}{{{\delta }U_{o} }},\bar{v}_{p} {{ = }}\frac{{\overset{\lower0.5em\hbox{$\smash{\scriptscriptstyle\frown}$}}{v} _{p} }}{{{\delta }U_{o} }},\overline{h} {{ = }}\frac{H}{{a_{1} }},\,\,\,{\beta }{{ = }}\frac{{a_{2} }}{{a_{1} }}, \hfill \\   \overline{u}  = \frac{{{{\overset{\lower0.5em\hbox{$\smash{\scriptscriptstyle\frown}$}}{u} }}}}{{u_{o} }},\,\,\,\,\overline{\mu } _{s}  = \frac{{\mu _{s} }}{{\mu _{o} }},\,\,\,\,\bar{p}{{ = }}\frac{{a{\delta }\overset{\lower0.5em\hbox{$\smash{\scriptscriptstyle\frown}$}}{p} }}{{U_{o} \mu _{s} }},{\bar{\Phi }}{{ = }}\frac{{{\overset{\lower0.5em\hbox{$\smash{\scriptscriptstyle\frown}$}}{\Phi } }}}{{\overset{\lower0.5em\hbox{$\smash{\scriptscriptstyle\frown}$}}{\zeta } }},{\bar{\psi }}{{ = }}\frac{{{\overset{\lower0.5em\hbox{$\smash{\scriptscriptstyle\frown}$}}{\psi } }}}{{a_{1} U_{o} }},{\delta }{{ = }}\frac{{a_{1} }}{{\lambda }},{{   U}}_{{HS}} {{ = }}\frac{{ - {\varepsilon \overset{\lower0.5em\hbox{$\smash{\scriptscriptstyle\frown}$}}{\zeta } }\overrightarrow {E} _{{\overset{\lower0.5em\hbox{$\smash{\scriptscriptstyle\frown}$}}{\zeta } }} }}{{U_{o} \mu _{s} }},{{ }}\,\, \hfill \\   m{{ = }}a_{1} ez\sqrt {\frac{{2n_{o} }}{{{\varepsilon }B_{o} T^{'} }}} ,{{  \text{M} = }}a_{1} B_{o} \sqrt {\frac{{\sigma }}{{\mu _{s} }}} ,\,\,\,\,B_{r}  = \frac{{\mu _{o} u^{{ * 2}} }}{{k(T_{1}  - T_{o} )}},\,\,\,\,R_{d}  = \frac{{16\sigma ^{ * } }}{{3k^{ * } }},\,\,\,\,\,\,\bar{T}^{ * }  = \frac{{\overset{\lower0.5em\hbox{$\smash{\scriptscriptstyle\frown}$}}{T}  - T_{o} }}{{T_{1}  - T_{o} }}. \hfill \\  \end{gathered}  \right\}  $$

By using the Eq. (17) into Eqs. (10)–(16), we get the following form of the dimensionless problem

18 $$ \frac{{\partial {\text{u}}_{f} }}{\partial \zeta } + \frac{{\partial v_{f} }}{{\partial\upeta }} = 0\;, $$

19 $$ \frac{\partial \left( p \right)}{{\partial (\zeta )}} = \frac{1}{{1 + \lambda_{1} }} \cdot \frac{{\partial^{2} {\text{u}}_{f} }}{{\partial\upeta ^{2} }} + \frac{{{\text{C}}S\left( {{\text{u}}_{p} - {\text{u}}_{f} } \right)a^{2} }}{{{(1 - }{\text{C}}{)}\mu_{s} }} - \left( {\frac{{M^{2} }}{{{(1 - }{\text{C}}{)}}}} \right){\text{u}}_{f} - \frac{\Phi }{\upvarepsilon } \cdot \frac{{m^{2} {\text{U}}_{HS} }}{{{(1 - }{\text{C}}{)}}}, $$

20 $$ \frac{{\partial {\text{u}}_{p} }}{\partial \zeta } + \frac{{\partial v_{p} }}{{\partial\upeta }} = 0, $$

21 $$ \frac{{\mu_{s} }}{{a\updelta \uplambda }}\frac{\partial p}{{\partial \zeta }}{ = }S\left( {{\text{u}}_{f} - {\text{u}}_{p} } \right), $$

22 $$ \left( {1 - R_{d} } \right)\frac{{\partial^{2} T^{ * } }}{{\partial \eta^{2} }} + B_{r} \left( {\frac{{\mu_{s} }}{{1 - \lambda_{1} }}\left( {\frac{\partial u}{{\partial \eta }}} \right)^{2} } \right) = 0. $$

(i) 23 $$ u_{f} = 0; \, \quad When \,\upeta = h_{g} (\zeta ), $$
(ii) 24 $$ u_{f} = 0;\quad When \,\upeta = - h_{g} (\overset{\lower0.5em\hbox{$\smash{\scriptscriptstyle\frown}$}}{\zeta } ). $$

The dimensionless form of Eq. (1) is

25 $$ h_{g} (\zeta ) = \left\{ {\begin{array}{*{20}l}    {1 - {{\upbeta}}\sin ^{2} \left( {\pi \zeta } \right)} \hfill & {{\text{When 0}}.5 < \zeta  < 1.5,} \hfill  \\    {0.5;} \hfill & {{\text{Othwewise}}.} \hfill  \\   \end{array} } \right. $$

After basic manipulation, the electro-osmotic potential function Φ(η), given in Eq. (19), can be obtained as

26 $$ {\Phi (\eta ) = }\frac{{\cosh (m\upeta )}}{{\cosh (mh_{g} )}}. $$

Using Eq. (26) in Eq. (19), we have

27 $$ \frac{1}{{1 + \lambda_{1} }} \cdot \frac{{\partial^{2} {\text{u}}_{f} }}{{\partial\upeta ^{2} }} - \left( {\frac{{M^{2} }}{{{(1 - }{\text{C}}{)}}}} \right){\text{u}}_{f} + \frac{{a^{2} {\text{C}}S\left( {{\text{u}}_{p} - {\text{u}}_{f} } \right)}}{{{(1 - }{\text{C}}{)\mu }_{s} }} - \left( {\frac{{{\text{m}}^{2} {\text{U}}_{HS} }}{{{(1 - }{\text{C}}{)}}}} \right) \, \frac{{\cosh (m\upeta )}}{{\cosh (mh_{g} )}} = \frac{\partial p}{{\partial \zeta }}. $$

From Eq. (21), we can write

28 $$ {\text{u}}_{p} { = }\;{\text{u}}_{f} - \left( {\frac{{\upmu _{s} }}{{a\updelta \uplambda S}}} \right)\frac{\partial p}{{\partial \zeta }}. $$

Substituting Eq. (28) into Eq. (27), we get

29 $$ \frac{{\partial^{2} {\text{u}}_{f} }}{{\partial\upeta ^{2} }} - A_{1} {\text{u}}_{f} = A_{2} P + A_{3} \cosh (m\upeta ). $$

By solving the above equation, we get the following form of required quantities

30 $$ \left. \begin{aligned} u_{f} = & \left( { - \frac{{P\left( {1 + \lambda_{1} } \right)\pi_{d} + \left( {\left( {1 + \lambda_{1} } \right)\cosh \left[ {h_{g} m} \right] + m\beta \sinh \left[ {h_{g} m} \right]} \right)\pi_{e} }}{{2\left( {\left( {1 + \lambda_{1} } \right)\cosh \left[ {h_{g} \sqrt {\pi_{a} } } \right] + \beta \sinh \left[ {h_{g} \sqrt {\pi_{a} } } \right]\sqrt {\pi_{a} } } \right)}}} \right) \\ & \times \left( {\cosh \left[ {\sqrt {\pi_{a} } \eta } \right] + \sinh \left[ {\sqrt {\pi_{a} } \eta } \right]} \right) + \left( {\cosh \left[ {\sqrt {\pi_{a} } \eta } \right] - \sinh \left[ {\sqrt {\pi_{a} } \eta } \right]} \right) \\ & \times \left( { - \frac{{P\left( {1 + \lambda_{1} } \right)\pi_{d} + \left( {\left( {1 + \lambda_{1} } \right)\cosh \left[ {h_{g} m} \right] + m\beta \sinh \left[ {h_{g} m} \right]} \right)\pi_{e} }}{{2\left( {\left( {1 + \lambda_{1} } \right)\cosh \left[ {h_{g} \sqrt {\pi_{a} } } \right] + \beta \sinh \left[ {h_{g} \sqrt {\pi_{a} } } \right]\sqrt {\pi_{a} } } \right)}}} \right) + \pi_{d} P + \pi_{e} \cosh \left[ {m\eta } \right]. \\ \end{aligned} \right\} $$

31 $$ \left. \begin{aligned} u_{p} = & \left( { - \frac{{P\left( {1 + \lambda_{1} } \right)\pi_{d} + \left( {\left( {1 + \lambda_{1} } \right)\cosh \left[ {h_{g} m} \right] + m\beta \sinh \left[ {h_{g} m} \right]} \right)\pi_{e} }}{{2\left( {\left( {1 + \lambda_{1} } \right)\cosh \left[ {h_{g} \sqrt {\pi_{a} } } \right] + \beta \sinh \left[ {h_{g} \sqrt {\pi_{a} } } \right]\sqrt {\pi_{a} } } \right)}}} \right) \\ & \times \left( {\cosh \left[ {\sqrt {\pi_{a} } \eta } \right] + \sinh \left[ {\sqrt {\pi_{a} } \eta } \right]} \right) + \left( {\cosh \left[ {\sqrt {\pi_{a} } \eta } \right] - \sinh \left[ {\sqrt {\pi_{a} } \eta } \right]} \right) \\ & \times \left( { - \frac{{P\left( {1 + \lambda_{1} } \right)\pi_{d} + \left( {\left( {1 + \lambda_{1} } \right)\cosh \left[ {h_{g} m} \right] + m\beta \sinh \left[ {h_{g} m} \right]} \right)\pi_{e} }}{{2\left( {\left( {1 + \lambda_{1} } \right)\cosh \left[ {h_{g} \sqrt {\pi_{a} } } \right] + \beta \sinh \left[ {h_{g} \sqrt {\pi_{a} } } \right]\sqrt {\pi_{a} } } \right)}}} \right)\; \\ & + \pi_{d} P + \pi_{e} \cosh \left[ {m\eta } \right] - \left( {\frac{{\mu_{s} }}{a\delta \lambda S}} \right)P. \\ \end{aligned} \right\} $$

32 $$ \begin{aligned} T = & c_{1} + \eta c_{2} + \frac{1}{{4\left( {1 + R_{d} } \right)\left( {m^{2} - \pi_{a} } \right)\left( {1 + \lambda_{1} } \right)}}B_{r} \\ &\left( \begin{aligned} & - \frac{1}{2}m^{2} \left( {R_{3}^{2} \cosh \left[ {2\eta \sqrt {\pi_{a} } } \right] + R_{4}^{2} \cosh \left[ {2\eta \sqrt {\pi_{a} } } \right] + 2R_{3} R_{4} \sinh \left[ {2\eta \sqrt {\pi_{a} } } \right]} \right) + \frac{1}{2} \\ &\left( {R_{3}^{2} + R_{4}^{2} } \right)\cosh \left[ {2\eta \sqrt {\pi_{a} } } \right]\pi_{a} + R_{3} R_{4} \sinh \left[ {2\eta \sqrt {\pi_{a} } } \right]\pi_{a} - \left( {R_{3}^{2} - R_{4}^{2} } \right)\eta^{2} \pi_{a}^{2} \\ & + \frac{{8m^{3} \sinh \left[ {m\eta } \right]\left( {R_{4} \cosh \left[ {\eta \sqrt {\pi_{a} } } \right] + R_{3} \sinh \left[ {\eta \sqrt {\pi_{a} } } \right]} \right)\sqrt {\pi_{a} } \pi_{e} }}{{\left( { - m + \sqrt {\pi_{a} } } \right)\left( {m + \sqrt {\pi_{a} } } \right)}} \\& - \frac{{16m^{2} \cosh \left[ {m\eta } \right]\left( {R_{3} \cosh \left[ {\eta \sqrt {\pi_{a} } } \right] + R_{4} \sinh \left[ {\eta \sqrt {\pi_{a} } } \right]} \right)\pi_{a} \pi_{e} }}{{\left( { - m + \sqrt {\pi_{a} } } \right)\left( {m + \sqrt {\pi_{a} } } \right)}} \\ & + \frac{{8m\sinh \left[ {m\eta } \right]\left( {R_{4} \cosh \left[ {\eta \sqrt {\pi_{a} } } \right] + R_{3} \sinh \left[ {\eta \sqrt {\pi_{a} } } \right]} \right)\pi_{a}^{3/2} \pi_{e} }}{{\left( { - m + \sqrt {\pi_{a} } } \right)\left( {m + \sqrt {\pi_{a} } } \right)}} + m^{4} \eta^{2} \pi_{a}^{2} \\ & - \frac{1}{2}m^{2} \cosh \left[ {2m\eta } \right]\pi_{e}^{2} + \frac{1}{2}\cosh \left[ {2m\eta } \right]\pi_{a} \pi_{e}^{2} + m^{2} \eta^{2} \pi_{a} \left( {R_{3}^{2} - R_{4}^{2} - \pi_{e}^{2} } \right) \\ \end{aligned} \right)\mu_{s} . \\ \end{aligned} $$

The volumetric flow rate is

33 $$ \left. \begin{gathered} Q = \left( \begin{gathered} P\left( {h_{g} \left( {1 + \lambda_{1} } \right)\cosh \left[ {h\sqrt {\pi_{a} } } \right] + \frac{{\sinh \left[ {h_{g} \sqrt {\pi_{a} } } \right]\left( { - 1 - \lambda_{1} + h_{g} \beta \pi_{a} } \right)}}{{\sqrt {\pi_{a} } }}} \right)\pi_{e} \hfill \\ + \frac{{\left( \begin{gathered} - \sinh \left[ {h_{g} \sqrt {\pi_{a} } } \right]\left( {m\left( {1 + \lambda_{1} } \right)\cosh \left[ {h_{g} m} \right] + \beta \sinh \left[ {h_{g} m} \right]\left( {m^{2} - \pi_{a} } \right)} \right) \hfill \\ + \left( {1 + \lambda_{1} } \right)\cosh \left[ {h_{g} \sqrt {\pi_{a} } } \right]\sinh \left[ {h_{g} m} \right]\sqrt {\pi_{a} } \hfill \\ \end{gathered} \right)\pi_{e} }}{{m\sqrt {\pi_{a} } }} \hfill \\ \end{gathered} \right) \hfill \\ /\left( {\left( {1 + \lambda_{1} } \right)\cosh \left[ {h_{g} \sqrt {\pi_{a} } } \right] + \beta \sinh \left[ {h_{g} \sqrt {\pi_{a} } } \right]\sqrt {\pi_{a} } } \right) + h_{g} P\pi_{d} - \frac{{P\sinh \left[ {h_{g} \sqrt {\pi_{a} } } \right]\pi_{a} }}{\begin{gathered} \left( {1 + \lambda_{1} } \right)\cosh \left[ {h_{g} \sqrt {\pi_{a} } } \right]\sqrt {\pi_{a} } \hfill \\ + \beta \sinh \left[ {h_{g} \sqrt {\pi_{a} } } \right]\pi_{a} \hfill \\ \end{gathered} } \hfill \\ - \frac{{P\lambda_{1} \sinh \left[ {h_{g} \sqrt {\pi_{a} } } \right]\pi_{d} }}{{\left( {1 + \lambda_{1} } \right)\cosh \left[ {h_{g} \sqrt {\pi_{a} } } \right]\sqrt {\pi_{a} } + \beta \sinh \left[ {h_{g} \sqrt {\pi_{a} } } \right]\pi_{a} }} + \frac{{\sinh \left[ {h_{g} m} \right]\pi_{e} }}{m} \hfill \\ - \frac{{\cosh \left[ {h_{g} m} \right]\pi_{e} }}{{\left( {1 + \lambda_{1} } \right)\coth \left[ {h_{g} \sqrt {\pi_{a} } } \right]\sqrt {\pi_{a} } + \beta \pi_{a} }} \hfill \\ - \frac{{\lambda_{1} \cosh \left[ {h_{g} m} \right]\pi_{e} }}{{\left( {1 + \lambda_{1} } \right)\coth \left[ {h_{g} \sqrt {\pi_{a} } } \right]\sqrt {\pi_{a} } + \beta \pi_{a} }} - \frac{{m\beta \sinh \left[ {h_{g} m} \right]\pi_{e} }}{{\left( {1 + \lambda_{1} } \right)\coth \left[ {h_{g} \sqrt {\pi_{a} } } \right]\sqrt {\pi_{a} } + \beta \pi_{a} }} - \frac{{h_{g} P\mu_{s} }}{aS\delta \lambda }. \hfill \\ \end{gathered} \right\}, $$

The pressure is obtained from the above equation which is given by

34 $$ \left. {P = \frac{{\left( {aS\delta \lambda \left( \begin{gathered} 2m\left( {\left( {1 + \lambda_{1} } \right)\cosh \left[ {h_{g} m} \right] + m\beta \sinh \left[ {h_{g} m} \right]} \right)\pi_{e} \hfill \\ + \left( {1 + \lambda_{1} } \right)\coth \left[ {h_{g} \sqrt {\pi_{a} } } \right]\sqrt {\pi_{a} } \left( {mQ - 2\sinh \left[ {h_{g} m} \right]\pi_{e} } \right) \hfill \\ + \beta \pi_{a} \left( {mQ - 2\sinh \left[ {h_{g} m} \right]\pi_{e} } \right) \hfill \\ \end{gathered} \right)} \right)}}{{\left( \begin{gathered} 2amS\delta \lambda \left( { - 1 - \lambda_{1} + h_{g} \left( {1 + \lambda_{1} } \right)\coth \left[ {h_{g} \sqrt {\pi_{a} } } \right]\sqrt {\pi_{a} } + h_{g} \beta \pi_{a} } \right)\pi_{d} \hfill \\ - h_{g} m\left( {\left( {1 + \lambda_{1} } \right)\coth \left[ {h_{g} \sqrt {\pi_{a} } } \right] + \beta \sqrt {\pi_{a} } } \right)\sqrt {\pi_{a} } \mu_{s} \hfill \\ \end{gathered} \right)}}} \right\} $$

## Results and discussion

**Figure 2:**
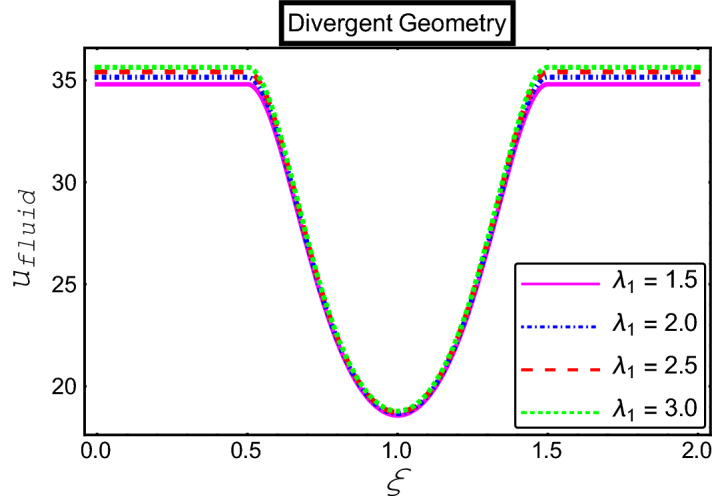
Impact of Jeffrey parameters on fluid velocity.

**Figure 3:**
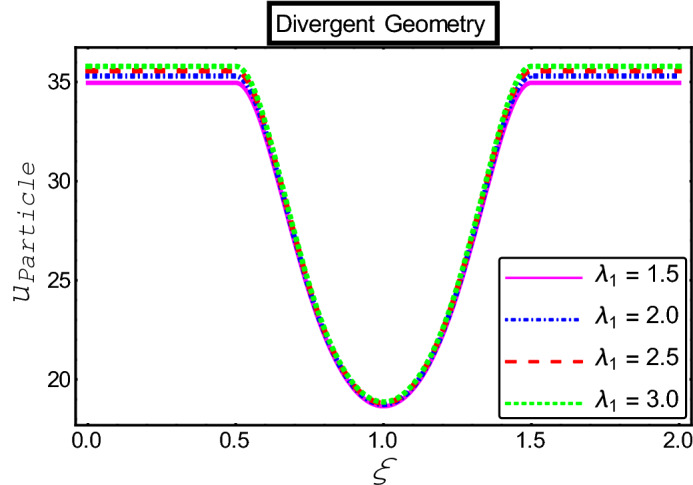
Impact of Jeffrey parameters on particle velocity.

**Figure 4:**
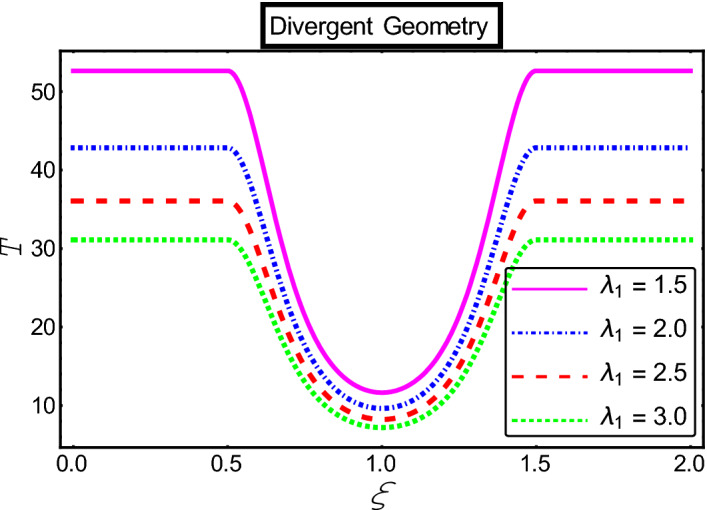
Impact of Jeffrey parameters on the temperature profile.

**Figure 5:**
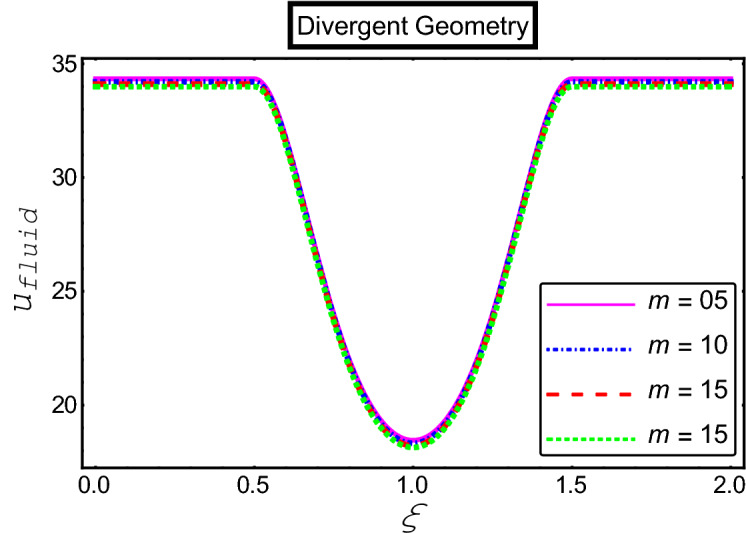
Impact of electro-osmotic parameter on fluid velocity.

**Figure 6:**
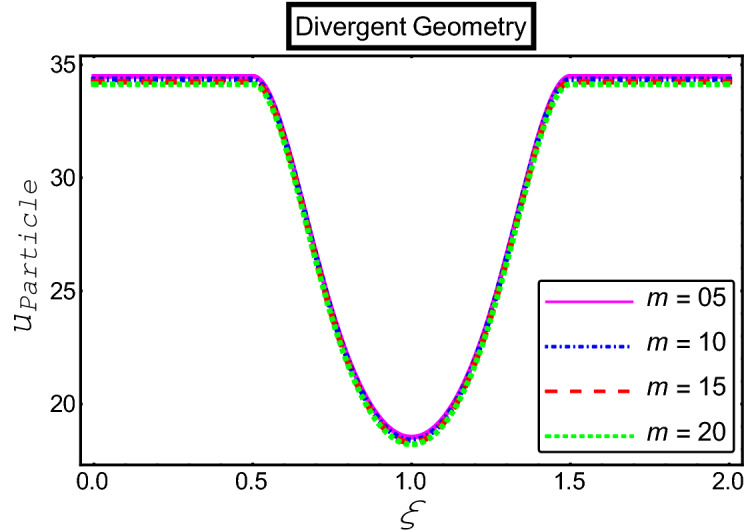
Impact of electro-osmotic parameter on the particle velocity.

**Figure 7:**
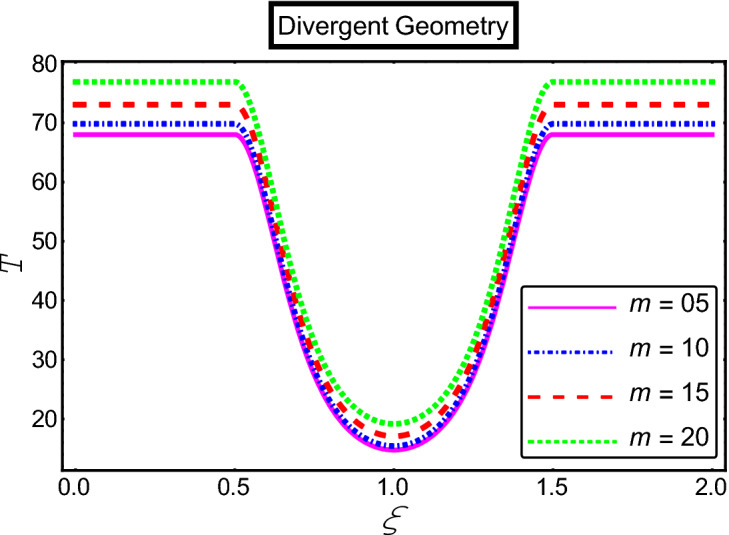
Impact of electro-osmotic parameter on the temperature profile.

**Figure 8:**
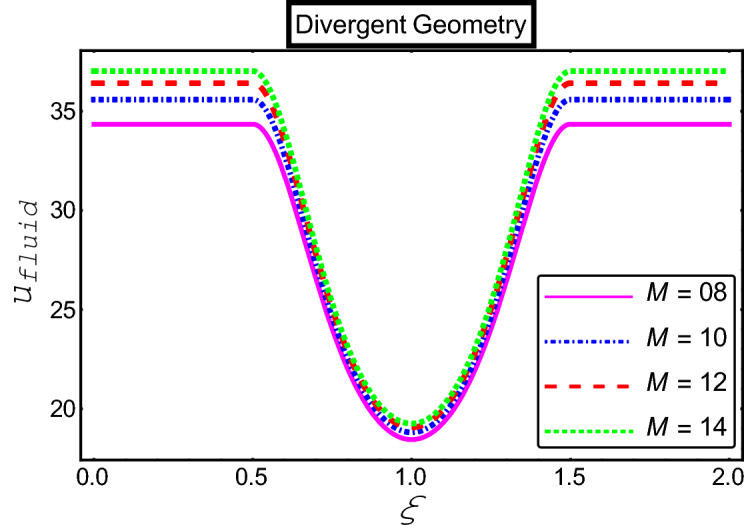
Impact of Hartmann numbers on fluid velocity.

**Figure 9:**
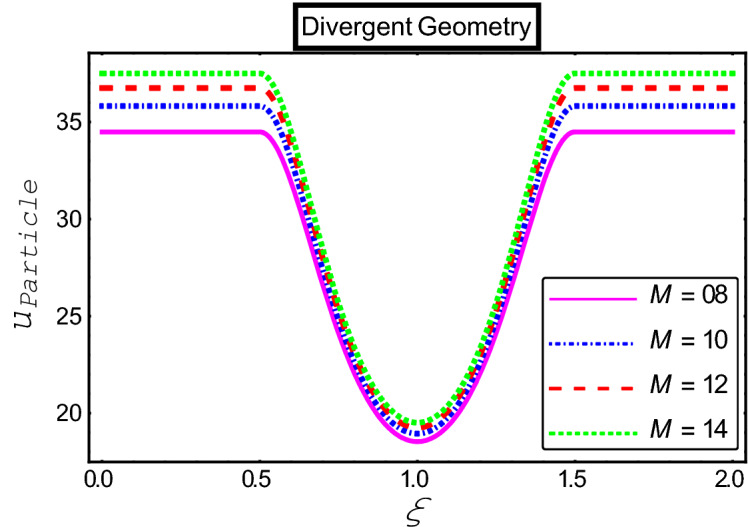
Impact of Hartmann numbers on particle velocity.

**Figure 10:**
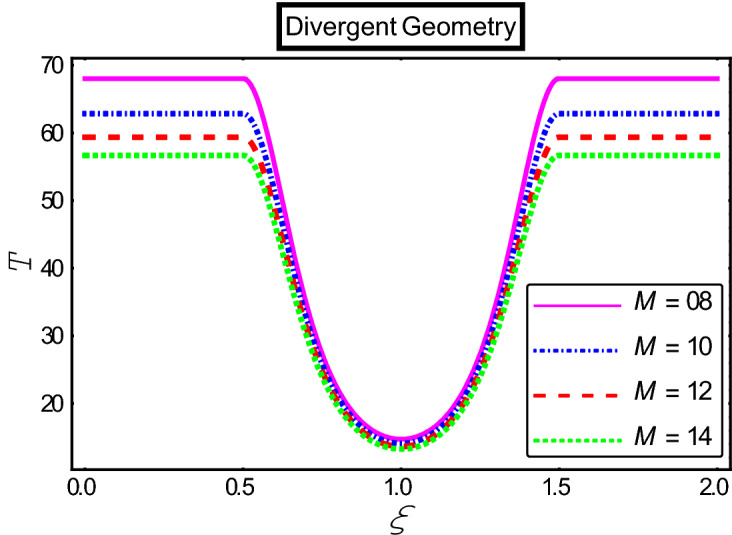
Impact of Hartmann numbers on the temperature profile.

**Figure 11:**
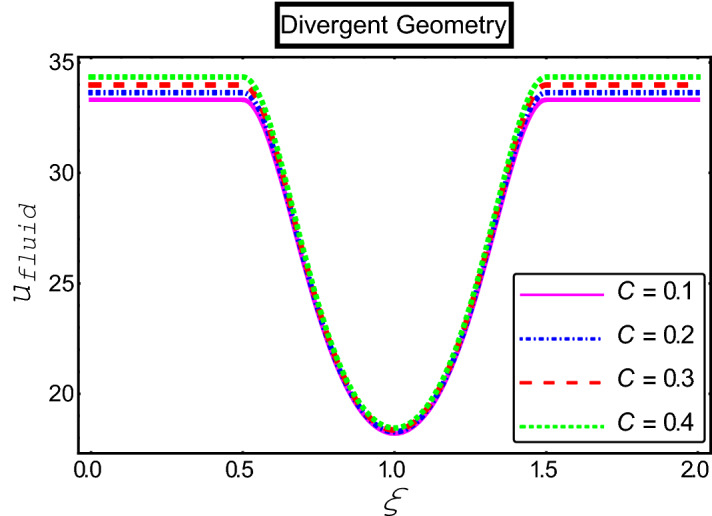
Impact of fluid velocity “*C*” parameters on fluid velocity.

**Figure 12:**
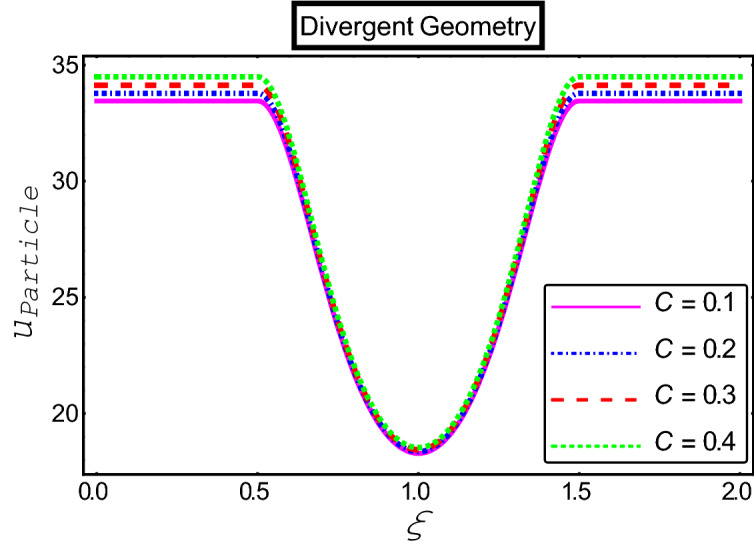
Impact of fluid velocity “*C*” parameters on particle velocity.

**Figure 13:**
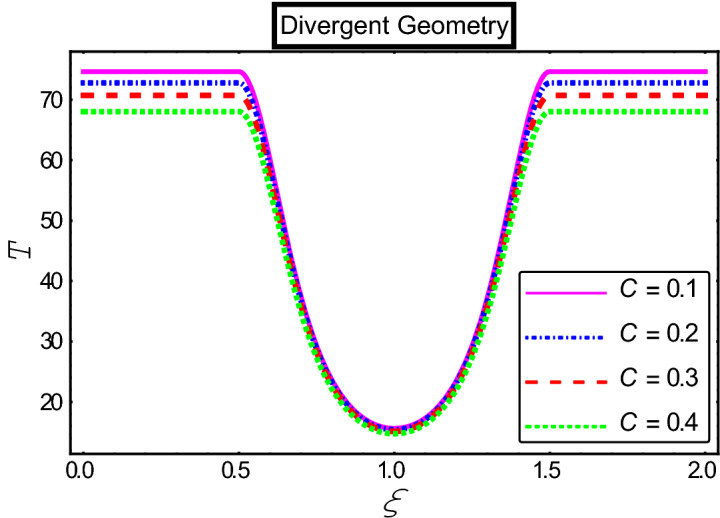
Impact of fluid velocity “*C*” parameters on the temperature profile.

**Figure 14:**
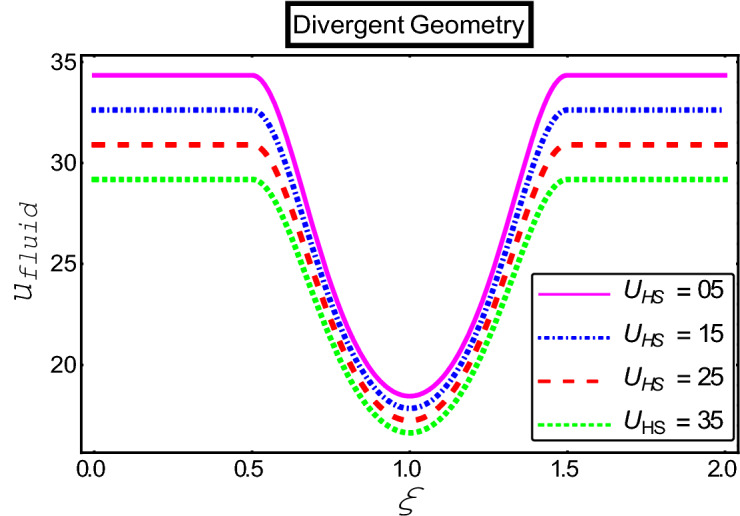
Impact of Helmholtz–Smoluchowski parameter on fluid velocity.

**Figure 15:**
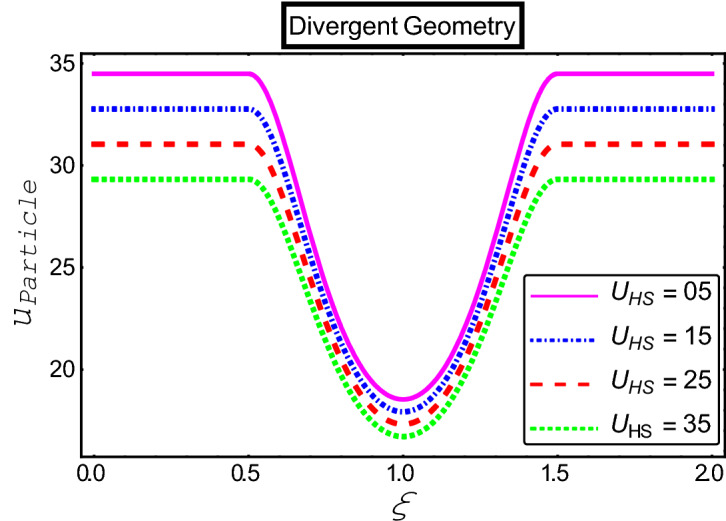
Impact of Helmholtz–Smoluchowski parameter on particle velocity.

**Figure 16:**
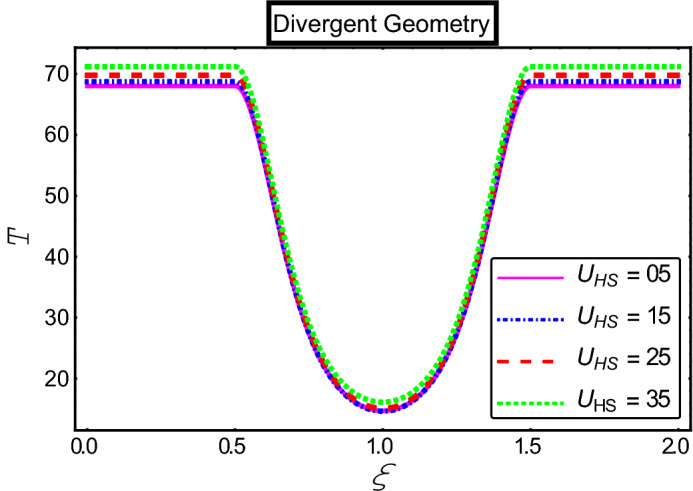
Impact of Helmholtz–Smoluchowski parameter on the temperature profile.

**Figure 17:**
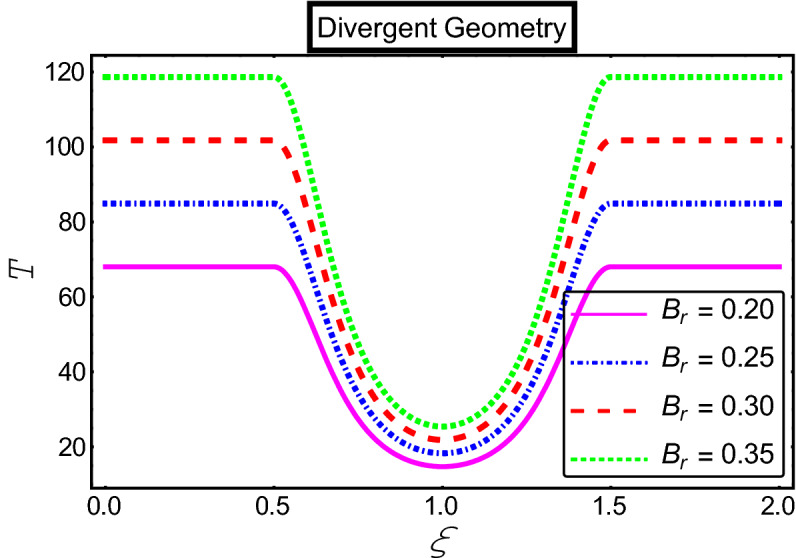
Impact of Brinkman number parameter on the temperature profile.

The objective of this section is to highlight the impact of important physical parameters on the velocity and temperature distribution through graphs. Figures 2, 3, 4, 5, 6, 7, 8, 9, 10, 11, 12, 13, 14, 15, 16 and 17 depict the influence of most significant parameters such as Jeffrey fluid parameter ${\lambda }_{1}$, electro-osmotic parameter *m,* Hartmann number *M, the* concentration of particles *C,* Helmholtz–Smoluchowski velocity ${U}_{HS}$, and Brinkman number ${B}_{r}$. Figures 2, 3 and 4 provide the influence of Jeffrey fluid parameter ${\lambda }_{1}$ on momentum and thermal profiles for an admissible range of the parameter. A vivid reduction in heat transfer is observed, in response to variation in the parameter. This is an opposite trend to the momentum profile of each phase. The effect of *m* on the velocity and temperature profiles is given in Figs. 4, 6 and 7. Unlike, the previous case velocity of each phase reduces while more thermal energy incorporates into the system by expediting the heat transfer. This phenomenon is the application of the electro-osmotic process which ionizes the charged groups on the surface or due to preferential adsorption of ions within the fluid. It acts transversely to the motion of the fluid due to Lorentz's force. Hartman number $M$ is a dimensionless number that corresponds to magnetic field induction. Figures 8, 9 and 10 highlights the impact of magnetic field on electro-osmotic flow Jeffrey fluid. One can notice that a reasonable enhancement in the behavior of fluid and particles velocity is observed as the strong magnetic field is applied. Variation in the number density of gold particles is sketched in Figs. 11, 12 and 13. With the addition of supplementary gold particles, the momentum of both phases gets more aggravated, due to their random motion in the divergent channel. However, the temperature profile shows a decline in Fig. 13. This suggests that heat is being transferred from the region of high temperature to a region of lower temperature. Helmholtz–Smoluchowski velocity ${U}_{HS}$ is another important emerging parameter. Helmholtz–Smoluchowski velocity has an effective contribution to the flow to determine the volumetric flow rate of viscoelastic fluids in microchannels. The impact of ${U}_{HS}$ on velocity and temperature profile are shown in Figs. 14, 15 and 16. It is observed that the increasing values of ${U}_{HS}$ decline the velocity profiles in both fluid and particle phases. However, it can be observed in Fig. 16 variation in ${U}_{HS}$ supports the temperature profile by increasing the force of friction between the adjacent layers of the base fluid. Finally, the influence of heat conduction from the wall on the viscous fluid is given in Fig. 17. It is noticed that more energy comes into the system due to slow down the process of conduction of heat by viscous dissipation when Brinkman number ${B}_{r}$ is varied. Hence, the temperature of multiphase flow rises.

## Concluded remarks

A closed-form solution is obtained for the heat of a non-Newtonian fluid suspended with gold particles. Electro-osmotic multiphase flow is analyzed in a divergent channel under the influence of viscous dissipation and thermal radiation. The most noteworthy observations catalog as:

- Jeffrey parameter corresponds to the rise of both velocity profiles.
- The electro-osmotic parameter *m* and Helmholtz–Smoluchowski velocity ${U}_{HS}$ act differently on thermal and momentum distribution.
- Additional gold particles expedite the flow of both phases.
- More energy is added to the system due to Brinkman number ${B}_{r}$.
